# Expression of SOX2 and OCT4 in odontogenic cysts and tumors

**DOI:** 10.1186/s13005-021-00283-1

**Published:** 2021-07-14

**Authors:** Ekarat Phattarataratip, Tarit Panitkul, Watunyoo Khodkaew, Pattarapong Anupuntanun, Jirapat Jaroonvechatam, Sirawit Pitarangsikul

**Affiliations:** 1 Department of Oral Pathology, Faculty of Dentistry, Chulalongkorn University, Henri-Dunant Road, Pathumwan, 10330 Bangkok, Thailand; 2 Faculty of Dentistry, Chulalongkorn University, Henri-Dunant Road, Pathumwan, 10330 Bangkok, Thailand

**Keywords:** SOX2, OCT4, Ameloblastoma, Ameloblastic fibroma, Odontogenic Keratocyst

## Abstract

**Background:**

Aberrant expression of stem cell markers has been observed in several types of neoplasms. This trait attributes to the acquired stem-like property of tumor cells and can impact patient prognosis. The objective of this study was to comparatively analyze the expression and significance of SOX2 and OCT4 in various types of odontogenic cysts and tumors.

**Methods:**

Fifty-five cases of odontogenic cysts and tumors, including 15 ameloblastomas (AM), 5 adenomatoid odontogenic tumors (AOT), 5 ameloblastic fibromas (AF), 5 calcifying odontogenic cysts (COC), 10 dentigerous cysts (DC) and 15 odontogenic keratocysts (OKC) were investigated for the expression of SOX2 and OCT4 immunohistochemically.

**Results:**

Most OKCs (86.7 %) and all AFs expressed SOX2 in more than 50 % of epithelial cells. Its immunoreactivity was moderate-to-strong in all epithelial cell types in both lesions. In contrast, SOX2 expression was undetectable in AOTs and limited to the ameloblast-like cells in a minority of AM and COC cases. Most DCs showed positive staining in less than 25 % of cystic epithelium. Significantly greater SOX2 expression was noted in OKC compared with DC or AM, and in AF compared with COC or AOT. OCT4 rarely expressed in odontogenic lesions with the immunoreactivity being mild and present exclusively in OKCs.

**Conclusions:**

SOX2 is differentially expressed in odontogenic cysts and tumors. This could be related to their diverse cells of origin or stages of histogenesis. The overexpression of SOX2 and OCT4 in OKC indicates the acquired stem-like property. Future studies should investigate whether the overexpression of OCT4 and SOX2 contributes to the aggressive behaviors of the tumors.

## Introduction

The SRY (sex determining region Y)-related HMG box (SOX) family of transcription factors consists of 20 protein members with highly conserved high mobility group (HMG) domains, responsible for specific DNA binding as well as nuclear localization. SOX2 is the most well-known and studied SOX family protein [1]. Its expression is present early during embryogenesis and involved in determining the neural lineage differentiation, as well as the structural development of selected endodermal and mesodermal origins [2]. In addition, SOX2, together with the transcription factors OCT4 (Octamer binding protein 4) and Nanog, helps preserve stemness property of pluripotential stem cells. Notably, the ectopic expression of SOX2, in cooperation with OCT4 and KLF4, could reprogram differentiated cells into induced pluripotential stem cells (iPSCs) [3].

SOX2-expressing stem cells can be detected in a variety of normal adult tissues, such as retina, stomach, testis, trachea, dermal papilla of hair follicle and dental epithelium [1]. Several studies noted the aberrant SOX2 expression in different types of benign and malignant neoplasms. This has been associated with the acquired stem-like property of tumor cells [4]. The impact of SOX2 on tumorigenesis is variable depending on tumor types. The increased SOX2 expression is associated with poor patient prognosis in cancers of the oral cavity [5], esophagus [2], colorectal [6], lung, breast [7], liver [8] and prostate [9], whereas in gastric cancer, worse clinical outcome is correlated with the diminished SOX2 expression in cancer cells [10, 11].

OCT4 is a transcription factor and regulator of the POU domain, involved in early embryogenesis and maintenance of embryonic stem cell pluripotency [12]. SOX2 works in complex with OCT4, forming an integrated network to sustain the self-renewal property of embryonic stem cells (ESC) and resist cellular differentiation [13].

Odontogenic cysts and tumors constitute a group of entities derived from remnants of the odontogenic apparatus of developing tooth. During normal odontogenesis, different types of odontogenic epithelium and odontogenic ectomesenchyme variably express SOX2 [14]. Its expression is observed in a time-dependent manner in ameloblasts, odontoblasts, dental papilla and dental follicle [15]. The dental lamina of developing human primary molar express SOX2, whereas no expression of this protein was detected in the Hertwig’s epithelial root sheath or epithelial rests of Malassez [16]. The presence of SOX2-positive stem cells in dental lamina indicates the epithelial competence for tooth development and supports the renewal of ameloblasts and other dental epithelial lineages [17, 18]. Overexpression of SOX2 in human dental pulp stem cells (DPSC) by retroviral infection has been shown to promote cell proliferation, migration, adhesion as well as the differentiation into odontoblasts [19, 20].

The pathogenesis of different types of odontogenic cysts and tumors are far from understood. They represent the most common jaw lesions with varying clinical characteristics and disease behavior. Dentigerous cyst (DC) and odontogenic keratocyst (OKC) are the two most common developmental odontogenic cysts. While DC poses excellent prognosis and rarely recurs, OKC demonstrates as high as 30 % recurrence rate following enucleation [21]. Within the group of epithelial odontogenic tumor, ameloblastoma (AM) is locally aggressive in nature, whereas adenomatoid odontogenic tumor (AOT) have limited growth potential with virtually no recurrence[22]. Calcifying odontogenic cyst (COC) is the cystic lesion showing ameloblastoma-like epithelium with low recurrence potential [23]. Ameloblastic fibroma (AF) is a true mixed odontogenic tumor derived from both the odontogenic epithelium and ectomesenchyme with variable prognosis [24].

A handful of studies investigated the role of SOX2 and OCT4 in this group of lesions [25–28]. The expression of SOX2 has been observed in the basal layer of DCs [27] and in the ameloblast-like cells as well as the stellate reticulum-like cells of AMs [16]. In addition, SOX2 was shown to be overexpressed in ameloblastic carcinomas and the strong nuclear immunohistochemical staining of SOX2 was associated with the high-grade transformation of AMs [27]. A genome-wide expression study showed the upregulation of SOX2 in OKCs, compare with AMs [26]. These data suggest that SOX2 is variably expressed in odontogenic cysts and tumors and may reflect their different histogenesis and potential stemness abilities. The expression of SOX2 in other odontogenic lesions, including AF, AOT and COC has not been previously defined. We hypothesize that SOX2 and OCT4 are differentially expressed in these odontogenic lesions. Therefore, the objective of this study was to comparatively analyze the pattern of SOX2 and OCT4 expression in various types of odontogenic cysts (DC, OKC, COC) and tumors (AM, AOT, AF).

## Materials and methods

### Tissue samples

**Fig. 1:**
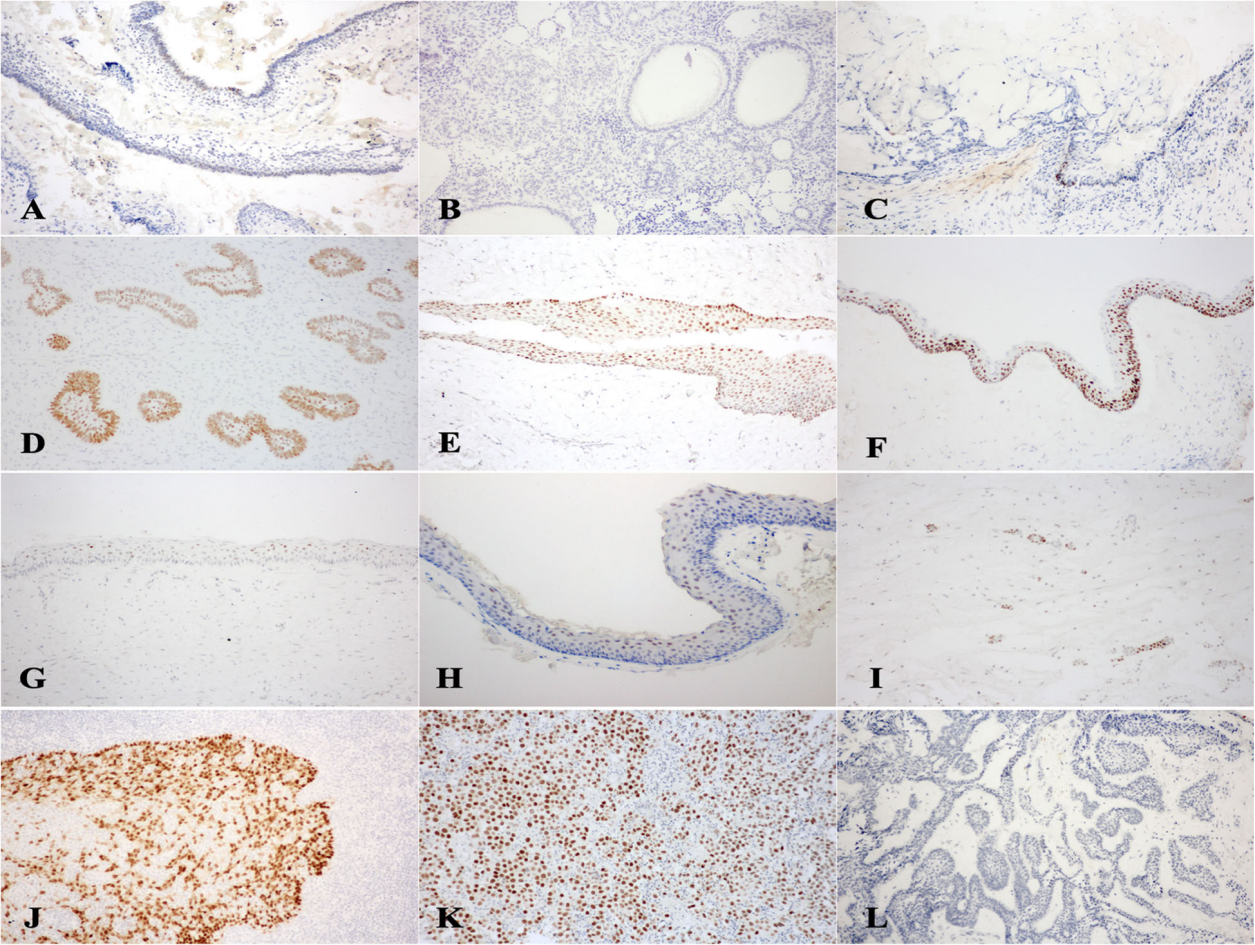
SOX2 and OCT4 expression in odontogenic cysts and tumors. AMs showed focal mild-to-moderate SOX2 staining limited to peripheral ameloblast-like cells (Level 1+) (**A**). SOX2 was undetectable in AOTs (Level 0) (**B**) and expressed minimally in a few ameloblast-like cells in COC (Level 1+) (**C**). In contrast, moderate-to-strong SOX2 expression was noted in all layers of odontogenic epithelium in most AFs (Level 4+) (**D**), DC (Level 3+) (**E**) and OKC (Level 4+) (**F**). Two cases of OKC showed Level 2 + immunoreactivity of SOX2 (**G**). OCT4 expressed exclusively in OKCs. In OCT4 positive cases, the staining was mild (intensity level +) and localized to the upper epithelial layers (Level 1+) (**H**). In normal dental follicles, rests of odontogenic epithelium expressed SOX2 at moderate (intensity level ++) staining (**I**). Tonsillar (**J**) and germinoma (**K**) specimens were used as positive controls for SOX2 and OCT4, respectively. Both represented the strong (intensity level +++) staining. Negative controls were stained with isotype-matched antibodies (**L**) and used as intensity level 0. (100X Magnification)

Fifty-five cases of odontogenic cysts and tumors, including 15 AMs, 5 AOTs, 5 AFs, 5 COCs, 10 DCs and 15 OKCs were included. All microscopic slides were reviewed to confirm the diagnoses based on the 2017 World Health Organization of Head and Neck Tumours (4th Edition) criteria. Sufficient clinical data was present in all cases. Patient information, including age, sex, and anatomical site, was recorded. Two cases of dental follicles were used to represent the corresponding normal tissues (Fig. 1I). The approval was obtained from the Head of the Department of Oral Pathology, Faculty of Dentistry, Chulalongkorn University to utilize the biopsy specimens for this study. The study was approved by the Human Research Ethics Committee at the Faculty of Dentistry, Chulalongkorn University. All methods were carried out in accordance with relevant guidelines and regulations.

### Immunohistochemical methods

The immunohistochemical staining was performed with Leica Microsystems Bond-Max Autostainer System as previously described [29]. The 5-µm thick specimen sections were deparaffinized at 60^o^C using 60-minute incubation with the Bond Dewax Solution. The antigen retrieval was performed by incubating slides with the Bond Epitope Retrieval Solution 2 for 30 min at 100^o^C, followed by 5-minute incubation with 3 % hydrogen peroxide.

Primary antibodies used were the rabbit polyclonal anti-SOX2 antibody (clone SP76, Cell Marque, Rocklin, CA) at 1:100 dilution and the mouse monoclonal ant-OCT4 antibody (clone sc-5279, Santa Cruz Biotechnology, Dallas, TX) at 1:300 dilution. The immunohistochemical procedure was performed using the Bond Polymer Refine Detection kit (Leica Microsystems), a 3-step indirect immunoperoxidase technique. The primary antibodies were applied for 40 min at room temperature, followed by 8 min incubation with the Post Primary Polymer and the Polymer Poly-HRP IgG, respectively. The sections were then incubated with diaminobenzidine for 4 min and counterstained with hematoxylin. The Bond Wash Solution was used to rinse between each step. As positive controls, tonsillar tissue (Fig. 1J) and germinoma (Fig. 1K) samples were used for SOX2 and OCT4 immunostainings, respectively. Negative controls were prepared using isotype-matched antibodies (Fig. 1L).

### Immunostaining assessment and statistical analysis

The immunohistochemical evaluation was performed and statistically analyzed using previously described criteria [30]. The positive immunoreactivity localized at the nucleus was evaluated. Overall, the percentage of positive cells was semi-quantitatively assessed and categorized into one of the following groups: 0 = no positive cells; 1 + = positive cells detected less than 25 %; 2 + = positive cells detected between 26 and 50 %; 3 + = positive cells detected between 51 and 75 % and 4 + = positive cells detected more than 75 %. The staining intensity was classified into 4 levels: level 0 = no staining; level + = mild staining; level ++ = moderate staining and level +++ = strong staining. All authors assessed and agreed upon the categorical immunostaining of each case.

The results were statistically analyzed using the IBM SPSS Statistics version 22 (IBM Corporation, NY) for Windows. The continuous variables were expressed as means ± standard deviation (SD). Comparative analyses of different levels of expression among groups were performed using Kruskal-Wallis test, followed by post-hoc pairwise comparison using the Bonferroni method. A P-value less than 0.05 was considered statistically significant.

## Results

Characteristics of 55 patients enrolled were presented in Table 1. The average age of AM, AOT, AF, COC, DC and OKC patients were 43.73 ± 19.01, 16.40 ± 8.38, 15.00 ± 7.21, 32.60 ± 13.52, 42.70 ± 16.23 and 37.13 ± 25.46 years, respectively. The male-to-female ratios of respective lesions were 1:1.1, 1:4, 1:4, 4:1, 1.5:1 and 4:1. Of the 15 AM cases, the histopathologic subtypes based on the predominant microscopic pattern were plexiform (6 cases), follicular (5 cases), acanthomatous (2 cases) and demoplastic (2 cases) patterns. All COC cases were of simple cystic type. Table 2 showed the levels of immunohistochemical staining of SOX2 and OCT4 in different types of odontogenic cysts and tumors. The staining intensity of distinct cell types in each tumor was detailed in Table 3.

**Table 1:** Patient characteristics

Tumors	Sex		Age		Location			
					Maxilla		Mandible	
	Male	Female	Mean ± SD	Range	Ant	Post	Ant	Post
AM (15)	7	8	43.73 ± 19.01	20–83	1	2	3	9
AOT (5)	1	4	16.40 ± 8.38	6–25	1	2	2	0
AF (5)	1	4	15.00 ± 7.21	8–25	0	2	1	2
COC (5)	4	1	32.60 ± 13.52	14–48	0	2	2	1
DC (10)	6	4	42.70 ± 16.23	11–64	3	3	0	4
OKC (15)	12	3	37.13 ± 25.46	7–84	0	6	3	6

**Table 2:** Levels of SOX2 and OCT4 expression in odontogenic cysts and tumors, based on the semi-quantitative assessment of percentage of positive cells. Analysis of comparison was performed using Kruskal-Wallis test

Stem cell markers	Odontogenic tumors	Immunohistochemical staining, n (%)					*P*-value
		Level 0	Level 1+	Level 2+	Level 3+	Level 4+	
SOX2	AM^a^	11 (73.3)	4 (26.7)	0	0	0	<0.001
	AOT^b^	5 (100)	0	0	0	0	
	AF^b,c^	0	0	0	3 (60)	2 (40)	
	COC^c^	4 (80)	1 (20)	0	0	0	
	DC^d^	1 (10)	6 (60)	0	3 (30)	0	
	OKC^a,d^	0	0	2 (13.3)	1 (6.7)	12 (80)	
OCT4	AM	15	0	0	0	0	0.365
	AOT	5	0	0	0	0	
	AF	5	0	0	0	0	
	COC	5	0	0	0	0	
	DC	10	0	0	0	0	
	OKC	13 (86.7)	2 (13.3)	0	0	0	

Lesion pairs labelled by letters a, b, c and d showed statistically significant differences in expression using post hoc pairwise comparison tests

**Table 3:** SOX2 and OCT4 staining intensity on different odontogenic cell types

Odontogenic leions	Type of odontogenic cells	SOX2	OCT4
AM (15)	Ameloblast-like cells	+/++	0
	Stellate reticulum-like cells	0	0
	Squamous cells	0	0
	Granular cells	0	0
AOT (5)	Spindle epithelial cells	0	0
	Ductal-like structures	0	0
	Whorled masses	0	0
AF (5)	Columnar epithelium	+++	0
	Stellate reticulum-like cells	++	0
	Dental papilla-like stroma	0	0
COC (5)	Ameloblast-like	+	0
	Stellate reticulum-like cells	0	0
	Ghost cells	0	0
DC (10)	Basal/parabasal epithelial cells	++	0
	Upper epithelial cell layers	++	0
OKC (15)	Basal/parabasal epithelial cells	+++	0
	Upper epithelial cell layers	+++	+

### SOX2

AM demonstrated a relatively low SOX2 expression level, with 73.3 % of cases did not express this protein and the remainders (26.7 %) expressed SOX2 in less than 25 % of neoplastic cells (Level 1+). The positive immunoreactivity was mild-to-moderate in intensity and only limited to a portion of ameloblast-like cells at the periphery of ameloblastic units (Fig. 1A). SOX2 expression was not detectable in all AOT (Fig. 1B) and 80 % of COC cases. One COC expressed SOX2 at mild intensity in less than 25 % of epithelial cells (Level 1+). The expression was localized in several ameloblast-like cells at the basal portion of the cystic lining (Fig. 1C).

In contrast, all AFs and most OKCs (86.7 %) expressed SOX2 in more than 50 % of odontogenic epithelial cells. The majority of AFs (60 %) expressed SOX2 between 50 and 75 % of odontogenic epithelial cells (Level 3+), and the remaining cases (40 %) expressed SOX2 in more than 75 % of odontogenic epithelial cells (Level 4+). The positive immunostaining was noted within the odontogenic epithelium of both the periphery and central portion of epithelial nests, showing strong and moderate staining intensity, respectively (Fig. 1D). The dental papilla-like stromal cells in AFs demonstrated no SOX2 expression.

Most DCs (60 %) demonstrate SOX2 expression in less than 25 % of cystic epithelial lining (Level 1+), followed by staining between 50 and 75 % (Level 3+, 30 %). The SOX2 immunoreactivity in DCs could be detected in all layers of the cystic lining at moderate staining intensity (Fig. 1E). 80 % of OKCs showed positive SOX2 immunostaining in more than 75 % of epithelial cells (Level 4+) (Fig. 1F), followed by the staining between 25 and 50 % (Level 2+, 13.3 %) (Fig. 1G). The strong staining intensity was present in all layers of the cystic epithelial linings.

The strong statistical difference in SOX2 expression (*P* < 0.001) was noted, when comparing among lesions. The post hoc pairwise comparison tests demonstrated that the expression of SOX2 was significantly higher in OKC than that of DC and AM. In addition, AF significantly expressed SOX2 more than COC and AOT.

### OCT4

Apart from OKCs, other odontogenic cysts and tumors studied did not express OCT4. The expression of OCT4 was detected in 2 cases of OKCs (13.3 %) and both cases showed the expression in less than 25 % of the cystic epithelial cells (Level 1+). The staining intensity was mild and limited to the upper epithelial layers with no expression at the basal or parabasal cells (Fig. 1H).

## Discussion

In this study, we report the expression of SOX2 and OCT4 in different types of odontogenic cysts and tumors. Overall, the immunoreactivity of each protein appears to be relatively uniform among cases within the same entity. OKC and AF demonstrate strong SOX2 expression, whereas most AM, COC and DC cases largely show minimal to no expression of this protein. No staining of SOX2 is observed in all AOT cases. In addition, the OCT4 expression is mostly absent in all entities except OKC.

Our data show that SOX2 is significantly overexpressed in the cystic epithelial lining of OKC, compared with that of DC. Among odontogenic cysts, OKC is unique for its clinically aggressive nature with the recurrence rate being varied from 2.5 to 62 % after surgery [31]. Previous studies have shown that OKCs consistently express higher PCNA and Ki-67 than other jaw cysts, indicating its inherently increased proliferative potential. The proliferating cells in OKC are noted within all layers of the cystic epithelial lining and not limited to the basal cells as in DC or radicular cyst [32]. In this study, we observe the equivalently strong SOX2 expression in all epithelial layers of OKC. In addition, OKC is the only odontogenic lesion showing evidence of OCT4 expression. These data suggest the inherent stem-like potential of OKC. This acquired property could play a role in the increased proliferative capacity of the epithelial linings and the unusually higher recurrence rate of OKCs than other jaw cysts.

The variation in SOX2 expression in these odontogenic lesions may also reflect the different cells of origin during disease development. Previous studies in developing tooth germs have illustrated the variable SOX2 expression during different stages of tooth development. The strong and diffuse SOX2 expression is noted early within the dental epithelium during bud stage. With further development, SOX2 expression becomes limited and localized in selected areas of enamel organ, such as the labial cervical loop in incisors or the lingual side of outer enamel epithelium, the cervical loop and inner enamel epithelium in molars [14]. These data indicate that SOX2 is differentially expressed in a time-specific manner during odontogenesis.

While it is generally accepted that DC is developed from the accumulation of fluid between the reduced enamel epithelium and the crown of an unerupted tooth, OKC is believed to be derived from the remnants of dental lamina. The pathogenesis of OKC is currently believed to involve *PTCH1* mutation, found in over 85 % of syndromic OKCs and as high as 84 % of sporadic cases when analyzing the cystic epithelial linings separated from connective tissue [33]. This creates a truncated, non-functional PTCH1 protein, allowing the constitutive signal transduction of Hedgehog (Hh) pathway, and subsequently leads to the uncontrolled cell growth and proliferation. Interestingly, in a mouse model, the upregulation of Hedgehog pathway arrests tooth development at bud stage [34], the phase at which SOX2 is uniformly expressed [14]. The enrichment of SOX2 within all epithelial layers of OKC could indicate the origin of OKC from SOX2-positive odontogenic epithelial cells within the arrested dental lamina or bud stage.

In contrast to OKC, our data showed that in AM, OCT4 is not detectable, and SOX2 is minimally expressed in a minority of cases. These findings are consistent with the previous genome-wide expression study, showing that SOX2 is significantly overexpressed in OKC, compared with AM [26]. In addition, we observe the localized expression of SOX2 solely in the ameloblast-like cells at the periphery of ameloblastic units. AM demonstrates the histopathologic features resemblance to that of developing enamel organ of the late bell stage. This limited expression SOX2 in AM may parallel to the findings of selective SOX2 localization during the later stage of normal odontogenesis. Regarding the pathogenesis of AM, *BRAF* mutation is found in approximately 60–80 % of this tumor and believed to play an essential role in its tumorigenesis. Together with our data, it appears that AM attains minimal stemness ability and that the locally aggressive nature of AM may be resulted from other genetic aberrations, not related to SOX2 or OCT4.

We also note the significant SOX2 overexpression in the odontogenic epithelium of AF, compared with those in AOT or COC. AF is a true mixed odontogenic tumor derived from both the odontogenic epithelium and ectomesenchyme. Microscopically, AF consists of small nests and strands of odontogenic epithelium resembled the rudimentary enamel organ, surrounded by the dental papilla-like ectomesenchyme. The pathogenesis of AF is poorly understood. A recent study showed that *BRAF* p.V600E mutation was present particularly in the mesenchymal component in a subset of AF [35]. We found a moderate-to-strong SOX2 expression within the odontogenic epithelium of AF, whereas the dental papilla-like connective tissue stroma was non-reactive. The overexpression of SOX2 in the epithelial component of AF may suggest the more primitive dental epithelium than those of AM, COC and AOT. A previous study reported that in dental papilla cells, SOX2 was absent during the bud stage, but became evident, following the formation of enamel and dentin [15]. In human natal teeth, the strong expression of SOX2 is detected in the pulp tissue [36]. Notably, a recent study reported that overexpressing SOX2 in dental pulp stem cells (DPSCs) help promote odontoblastic differentiation and enhance the production of dentin sialoprotein (DSPP) and dentin matrix protein-1 (DMP) [19]. The lack of SOX2 in dental papilla-like mesenchymal cells of AF may underlie its inability to differentiate towards odontoblasts and produce dental hard tissue in this lesion.

Even though we note a relatively uniform expression pattern among groups, this study is limited by a rather small sample size in certain groups of odontogenic lesions, and the unmatched age or sex of participants. This is primarily due to the rarity of some odontogenic entities. Future studies with the increased number of samples in various age groups could help reaffirm our findings. In addition, it could be of interest to further examine the molecular mechanisms underlying the upregulation of SOX2 in OKC and AF and the impact of the SOX2-associated stem-like property on the clinical progression of these lesions.

In conclusion, we report in the present study the differential SOX2 with limited OCT4 expression in odontogenic cysts and tumors. This variation in SOX2 expression could be attributable to their diverse cells of origin and stages of histogenesis. In addition, SOX2 is overexpressed in OKC and the epithelial portion of AF, suggesting the inherent stemness characteristic of these entities. Further studies should examine whether SOX2 and OCT4 play a part in the clinically aggressive nature of OKC.

## Data Availability

The datasets used and/or analyzed during the current study are available from the corresponding author on reasonable request.
